# Pseudo-color enhancement of CBCT images as a diagnostic tool for radicular cysts and periapical granulomas: A clinical feasibility study

**DOI:** 10.1007/s00784-025-06425-y

**Published:** 2025-07-03

**Authors:** Noha M. Elkersh, Mohamed G. Hassan, Osama Zakaria

**Affiliations:** 1 Department of Oral medicine, Periodontology, Oral diagnosis and Oral Radiology, Faculty of Dentistry, Alexandria University, Alexandria, Egypt; 2 Department of Orthodontics, Faculty of Dentistry, Assiut University, Assiut, Egypt; 3 Postdoctoral Research Associate, Division of Bone and Mineral Diseases, School of Medicine, Washington University, St. Louis, MO USA; 4 Department of Biomedical Dental Sciences Oral and maxillofacial division, College of Dentistry, Imam Abdulrahman Bin Faisal University, Dammam, Saudi Arabia

**Keywords:** Cone-beam CT, Digital eye, Periapical lesions, Pseudo color enhancement

## Abstract

**Objective:**

The aim of this work was to present a novel, non-invasive, and cost-effective technique to effectively distinguish between radicular cysts and periapical granulomas. This approach involves the application of pseudo-color enhancement to cone-beam CT imaging data, which has the potential to provide clinicians with valuable preliminary diagnostic information prior to more invasive investigative procedures.

**Materials and methods:**

The study involved a total of 57 cases, including both radicular cysts and periapical granulomas, with cone-beam CT scans obtained for each case. The DICOM data were imported into an open-access software (R2Gate), and a pseudo-color enhancement tool was then applied to visualize the color distribution patterns within each case. A scoring system was subsequently developed based on the observed color distribution characteristics. Two blind investigators independently assessed and classified each case according to their diagnostic findings. The statistical analysis involved the use of the independent t-test to compare the two groups with normal distributions, and the categorical data were represented as numbers and percentages. The significance level for the statistical findings was set at 5%.

**Results:**

The reliability of the intra-examiner reliability was evaluated and showed high agreement (0.965). With regard to the inter-examiner reliability, it revealed high agreement (0.930). A statistically significant difference (*p* = 0.002) was revealed between the two groups (cysts and granulomas) according to the scoring system. The results showed that all cystic lesions were categorized in score 2 while 29 of cases diagnosed granulomas histopathologically (out of 49) followed score 1.

**Conclusion:**

Within the settings of this study, it was possible to distinguish between radicular cysts and periapical granulomas with the pseudo-color enhancement of cone-beam CT images.

**Clinical relevance:**

To date, no studies have evaluated the validity of using pseudo-color enhancement, available in open-access cone-beam CT software, for differentiating between radicular cysts and periapical granulomas. This novel technique holds promise as a non-invasive and affordable approach to distinguish these two common periapical lesions.

**Supplementary Information:**

The online version contains supplementary material available at 10.1007/s00784-025-06425-y.

## Introduction

Apical pathosis is the development of chronic inflammation in periapical tissues as the result of bacterial overload of the teeth root canals [1]. The prolonged presence of microbes in the root canal system leads to their spread in the periapical tissues. The consequence of this spread depends on the host immune response either acute or chronic inflammation [2]. The acute inflammation symptoms include pain, sensitivity to pressure and elevation of the tooth while the chronic inflammation is symptomless and causes bone resorption. This silent bone resorption leads to the development of periapical lesions that appear radiographically as bone rarefaction [1].

The periapical lesions could be either granulomas or cysts. The former is more frequent (85% of periapical lesions) and consists of granulation tissues, cell infiltrates and a capsule of fibrous tissue [3]. The latter is less frequent and is formed of cyst wall of non-keratinized stratified squamous epithelium and cystic fluid of different proteins content [4]. The current widely accepted management of these periapical lesions is different. Granulomas may heal after non-surgical root canal treatment, while cysts are less likely to heal with this conservative treatment and needs further apical surgery. However, the apical surgery could not be used as a routine treatment of periapical lesions because of its invasiveness, liability of nerve damage, affection of soft tissue and surrounding teeth and increased treatment cost [5].

The importance of noninvasive techniques for the differential diagnosis of periapical lesions prior to treatment has thus increased to avoid unnecessary invasive treatment. These techniques involved high resolution Ultrasonography [6, 7], Dental MRI [1], Computed Tomography [8, 9] and Cone Beam Computed Tomography [5, 10–12]. However, most of these techniques are not available in dental clinics and considered to be non-affordable [1, 6–9]. Also, the use of Gray values in cone-beam CT to differentiate between them was found to be not reliable [5]. As physiological characteristics of the human eye require special color compensations [13].

Various color scales have been proposed to replace the gray scale in conventional radiography, as there are more detectable colors with varying hues, saturations, and brightness than there are perceptible shades of gray. The human visual system only registers differences in light intensity when exposed to different shades of gray, which leads to this logical conclusion. Adding color enhances perception [14]. Pseudo-color enhancement in digital periapical radiographs has been previously investigated in the detection of interproximal caries [15, 16], root fracture [17], in digital subtraction radiography [18] and in sinus mucosa pathological changes recently [19]. It was concluded that the color coded images were less influenced by the bias in the interpretation of any pathological changes [18]. This tool is freely available in some cone-beam CT viewer software and can be applied in cone-beam CT scans already performed prior to dental treatment without the need of additional costs or equipment.

To the best of our knowledge, no studies have evaluated the potential application of pseudo-color enhancement in the evaluation of periapical lesions in cone-beam CT. This study aimed to assess the clinical feasibility of an affordable, noninvasive radiographic technique using color enhancement to distinguish radicular cysts from periapical granulomas and to preliminarily validate its diagnostic utility.

## Materials and methods

### Study design

This study was retrospective. It was conducted at Oral and Maxillofacial Radiology Department, Faculty of Dentistry, Alexandria University after approval of the research Ethics Committee of the faculty (IRB Registration number: SCCREIRB-DNTSTRYALX-GU-001-180325-042).A consent was obtained from the patients prior to the study.

### Data collection

The sample size was determined based on a 95% confidence level to assess the accuracy of cone-beam CT in detecting periapical lesions. Uraba et al. [20] reported an overall cone-beam CT accuracy of 52.2%, with a 95% confidence interval (CI) of 33.4–59.8%. The required sample size was calculated to be 52 scans, which was increased to 57 to account for potential laboratory processing problems. Sample size was calculated using MedCalc Statistical Software version 19.0.5 (MedCalc Software bvba, Ostend, Belgium).

A retrospective data of total 57 cone-beam CT scans of periapical lesions was collected in the period between January 2022 and December 2024. Only Cases that have undergone histopathologic examination of the periapical lesions were included. The lesions associated teeth were endodontically treated. Taking a biopsy was justified by the diagnosis that these teeth were hopelessly non-restorable. For this reason, they were extracted as a part of patients treatment plan and histopathologic examination for the lesions was done as it is a routine process conducted at the university for any excised living tissues. Histopathological sampling was performed within two weeks after cone-beam CT imaging, minimizing the risk of lesion transformation during the interval. High quality cone-beam CT scans were enrolled in the study while the following cases were excluded from the study: cases without histopathological confirmation, prior surgical intervention in the region, low-quality cone-beam CT, incomplete cone-beam CT dataset, and presence of systemic or local conditions that may affect lesion appearance (e.g., bone dysplasias, cystic lesions unrelated to pulp pathology).

Cone-beam CT examination was performed with the machine (Carestream Health, CS 9300, Atlanta, GA, USA) at voxel size 0.09*0.09*0.09 mm, 84kvp, 5 mA and 10*10 cm Field of View (FOV) and Digital Imaging and Communication in Medicine (DICOM) dataset was exported and saved on an external hard drive.

According to the histopathologic examination 2 groups of periapical lesions were assigned, either radicular cysts or periapical granulomas. All data was collected and written in an excel sheet including patients age, gender, date of examination and histopathologic diagnosis and was kept with one of the investigators.

### Cone-beam CT examination and analysis

DICOM data was imported in R2Gate cone-beam CT viewer software (Megagen implants, Korea, Daegu, South Korea) according to the workflow illustrated in the supplementary figure.

R2 gate software is an open access free software designed by Megagen implant system mainly for computer guided implant surgeries and the digital eye tool is mostly used to predict primary implant stability. However, this tool is available to be applied in any DICOM data set.

The pseudo-color enhancement tool (Digital eye) was applied in each case. This tool includes 5 filters, one of them changes the gray scale image into colored one with four main colors (red, blue, yellow and green). The cuts used for color enhancement were carefully selected and assessed first to make sure they are free from beam hardening artifacts that could act as confounder.

**Fig. 1:**
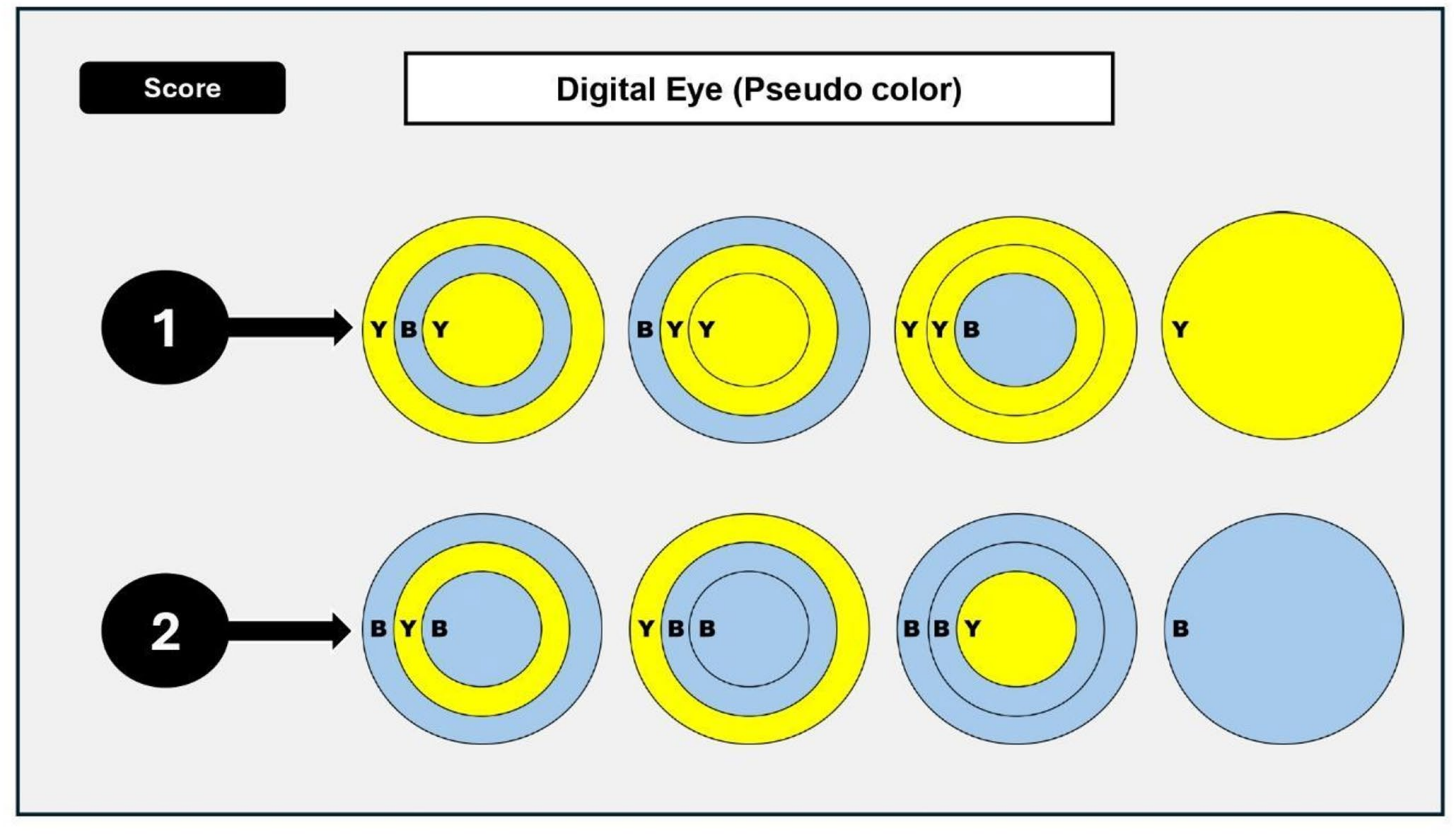
scoring system of color enhancement of periapical lesions

The distribution of the colors was assessed in each lesion in the axial and the sagittal view in three concentric parts: outer, middle and inner parts [19]. Two scores were suggested in this study based on the predominant color pattern across the lesion’s volume as follows and shown in Fig. 1.

Score 1: the whole lesion is yellow color coded/or.

one of the three concentric parts is blue color coded.

Score 2: the whole lesion is blue color coded/or.

one of the three concentric parts is yellow color coded.

The mentioned approach was exploratory and was adapted to capture potential heterogeneity in lesion architecture to identify whether pseudo-color patterns vary spatially within lesions.

Two investigators (M.G.H and N.M.E) with more than 7 years of experience in maxillofacial imaging analyzed each lesion blinded of the diagnosis of the lesions and categorized them according to the scoring mentioned above and agreement between them was evaluated as a first step in the study to ensure the reliability of the results. Observer 1 has reevaluated all the lesions after 2 week wash-up.

The third investigator (O.Z) categorized the findings according the histopathology (Cysts and granulomas) and tabulated all the data for the statistical analysis.

### Statistical analysis

Data was fed to the computer and analyzed using IBM SPSS software package version 20.0. (Armonk, NY: IBM Corp, released 2011). The distribution of the data was examined with the Shapiro Wilk test. An independent sample t-test was used for comparing two groups with normal distribution. Categorical data were represented as numbers and percentages. Chi-square test was applied to compare between two groups. Alternatively, Fisher Exact test was applied when more than 20% of the cells have expected count less than 5. Kappa (κ) for agreement was used. The significance of the obtained results was judged at the 5% level.

## Results

Assessment of demographic data was done and shown in (Table 1), the mean age (± SD) in cysts group was 34.38 ± 6.301 while in granulomas group was 34.04 ± 8.832 with no statistically significant difference between the 2 groups (Independent sample T-test).

Intra-examiner reliability was evaluated (Table 2) and revealed high agreement (0.965). Regarding the inter-examiner reliability, it revealed also high agreement between the 2 observers (0.930).

**Fig. 2:**
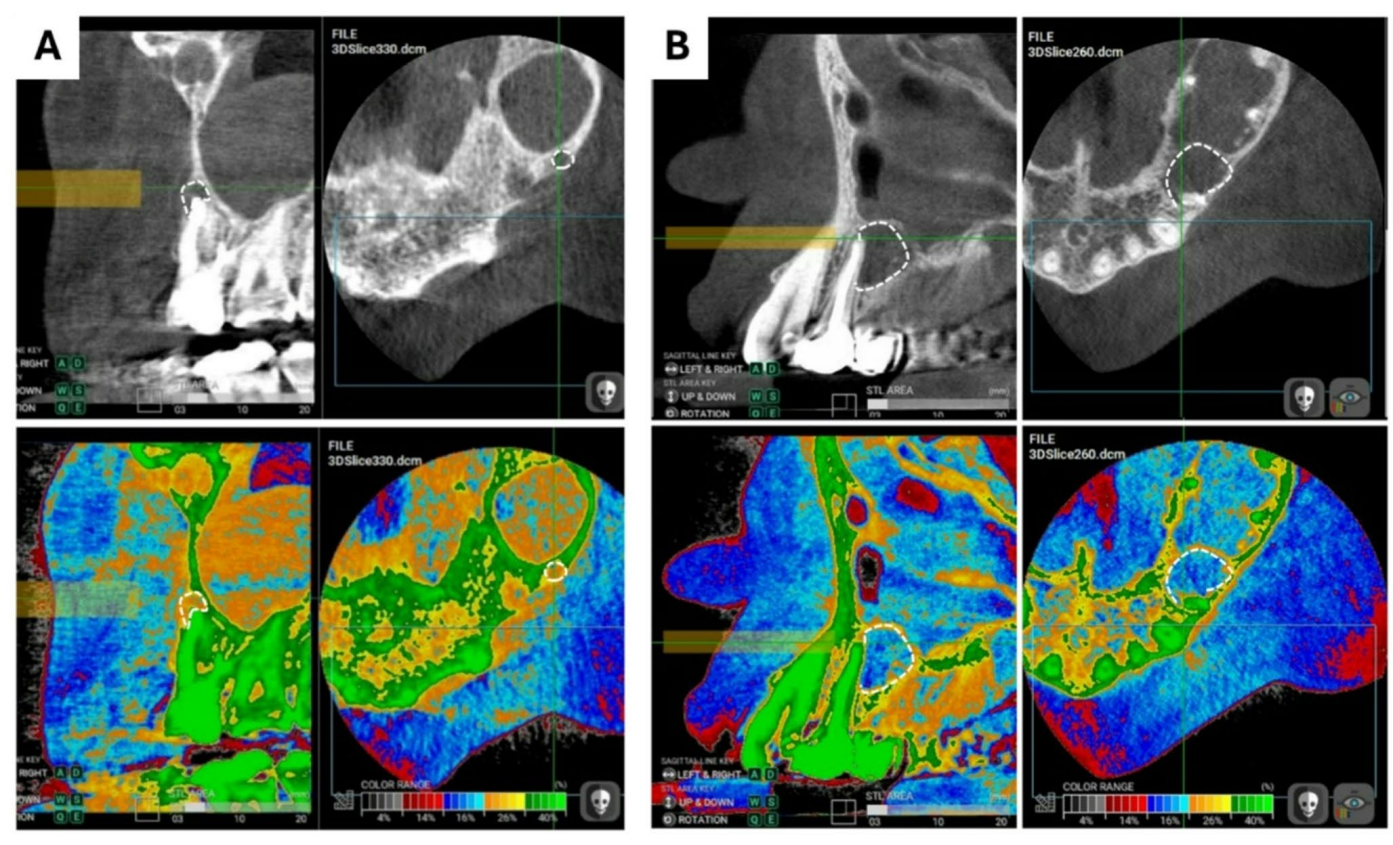
Top figures represent gray scaled images, Bottom represent color enhanced images of the same lesions (traced with dashed white line) where **A**: score 1 lesion (granuloma), **B**: score 2 lesion (cyst)

**Fig. 3:**
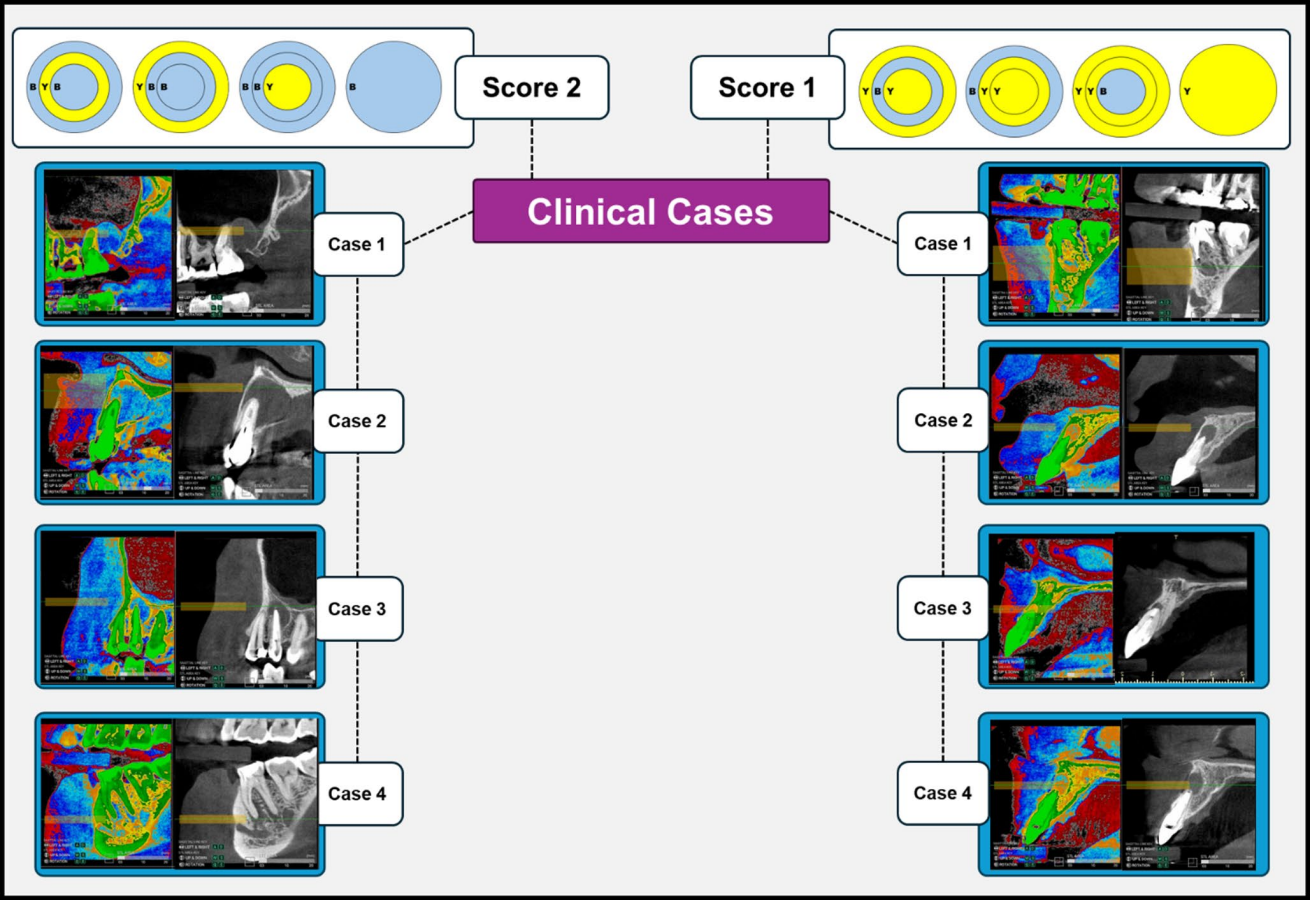
Clinical cases of radicular cysts (Left side) and periapical granulomas (Right side) with corresponding gray scaled images

The pairwise comparison between the 2 groups (cysts and granulomas) according to the scoring system was made, a statistically significant difference (*p* = 0.002) was revealed between them as shown in Table 3. It was found that all cystic lesions were categorized in score 2 while 29 (out of 49) of cases diagnosed as granulomas histopathologically followed score 1 (Figs. 2 and 3).

## Discussion

A noninvasive technique for differentiation between radicular cysts and periapical granulomas is crucial prior to the treatment in order to avoid unnecessary comprehensive management. Non-invasive diagnostic accuracy has clinical and academic value and offers a great potential for future precision treatment. Distinguishing cysts from granulomas can provide insights into lesion biology, chronicity, and treatment prognosis, which may influence long-term monitoring strategies or retreatment decisions in some of uncertain cases. Furthermore, regenerative endodontics are evolving. And in the near future, precise lesion characterization may become increasingly relevant to personalized treatment planning. For this reason, the validation of imaging-based techniques for histological inference advances diagnostic science and may reduce the necessity for invasive procedures in the future from a radiological perspective. Thus, our work represents technical proof-of-concept aimed at enhancing lesion visibility and interpretability, especially in settings with limited access to advanced imaging or specialist and when surgical access or histopathological confirmation are impractical. All the techniques found in the literature are either non affordable or inaccessible to dentists in their clinics [1, 6–9]. The current study aimed to introduce a new technique to differentiate between radicular cysts and periapical granulomas and evaluate the use of pseudo-color enhancement (digital eye) to differentiate between radicular cysts and periapical granulomas. Additionally, a scoring system was suggested to facilitate the differentiation of periapical lesions.

Gray scale radiographs can be converted into pseudo color by assigning a distinct color to a specified set of gray values. This generates a colored image that can leverage the property of human eyesight to discern thousands of color hues instead of only a restricted number of gray levels, especially that human eye detects only 32 out of 256 shades of gray. The benefit of assigning pseudo color to gray intensity values on a digital radiography image is that the human visual system can see color fluctuations more easily than gray [22]. Color can add a variable dimension that may outperform observers’ ability to absorb and process information. It has been determined that, in some situations, it could serve as a visual response mechanism trigger, resulting in a complete alteration of the original images [21].

Despite the evaluation of pseudo-color enhancement of radiographs in previous studies, its application was limited to digital two-dimensional radiographs [15–18]. None of them have evaluated the potential use of this tool for three-dimensional cone-beam CT images in periapical pathosis. Based on the results of the current clinical feasibility study, it was concluded that the pseudo-color enhancement tool (known as digital eye in R2Gate software) of cone-beam CT images could be used as a differentiating tool between radicular cysts and periapical granulomas without the need for additional equipment or costs.

Previous studies have evaluated the application of pseudo-color enhancement in the detection of proximal caries and demineralization [15, 16, 21]. The authors have assessed the presence of caries with and without the pseudo-color filter and have compared their findings to the histologic examination of the teeth as the gold standard. It was found that there is no significant difference between the gray scaled and colored image. These contradictory results could be explained by the fact that the observers were unfamiliar with the colored images from one side. From the opposite side, the images used in the previous studies were 2D images that lacked the third dimension, unlike 3D imaging modalities (cone-beam CT) that record the three spatial dimensions. Moreover, continuous improvements in software packages and their integrated color enhancement tools further optimize visual interpretation and clinical utility of pseudo-colorized images. However, another studies have assessed pseudo color enhancement in endodontic applications. The authors in a study [17] have evaluated fractured teeth with both the original images view (gray scale) and with different color enhancement filters provided with the software. It was found that color enhancement is superior to the original view in root fracture detection. Moreover, the accuracy of measurements of endodontic files was assessed [22] in single rooted teeth with software enhancement tools (pseudo color, emboss, sharpness and edge enhancement) and without the enhancement tools. The authors concluded that the least errors were associated with the pseudo color enhancement tool. In the literature, one study was found to evaluate the pseudo color enhancement in cone-beam CT images in order to diagnose pathological changes in maxillary sinus mucosa prior to implant surgery [19]. The findings of the study showed that pseudo color imaging could be used as a noninvasive diagnostic tool in implant surgery planning of maxillary posterior regions.

Even though the current study was positive, some limitations were encountered, such as the limited number of cases of radicular cysts. Balancing the groups would delay the release of a tool that could be refined and enhanced in the future to support different specialties like endodontics, pediatric dentistry, oral radiology and pathology. The key was to demonstrate the concept rigorously to guide larger studies including different lesions and incorporating higher level of accuracy. Therefore, future studies with a larger sample size and more balanced cohort are essential, and the need to validate the proposed color coding scoring system. This study should therefore be considered a foundational effort that demonstrates feasibility rather than conclusive diagnostic performance. Moreover, the scoring system used in the study may oversimplify complex cases, and a more nuanced or continuous scoring system may be needed in future studies. Another limitation is the variability in cone-beam CT hardware, software, voxel size, contrast settings, and monitor calibration that may influence pseudo-color visualization. We recommend a future multicenter study that considers several factors like reproducibility across different settings, and that efforts should be made to standardize image acquisition parameters and display calibration before clinical adoption can be expanded. Another point to be addressed is that pseudo-color enhancement is available in certain commercial software platforms and could be added as an adjunctive tool (within the limits of cone-beam CT’s known artifacts and variability), particularly for less experienced clinicians. However, its effectiveness may depend on standardized training and a familiarity with image interpretation. We also stress that it should complement, not replace, established diagnostic protocols.

One promising direction for future research is the possible integration of artificial intelligence in the classification of periapical lesions. It is possible to train machine learning algorithms on color-mapped datasets to support automated lesion classification. Pseudo-color imaging could serve as a complementary visualization layer for future AI-based diagnostic tools. Image-based decision support and deep learning are being used more and more in dental diagnostics. Color-enhanced features may provide better input dimensions for automated lesion classification models. In this context, our approach may support the development of personalized diagnostic pathways, enabling earlier and more tailored interventions based on lesion characteristics. This work is not immediately clinically applicable; however, we regard it as a preliminary step in the integration of accessible image-processing techniques into more comprehensive, AI-driven personalized care strategies.

## Conclusion

The findings of this study show that pseudo-color enhancement could be used as a noninvasive and affordable aid for the differentiation of radicular cysts and periapical granulomas prior to their treatment. However, the small number of cyst cases limits the extrapolation of these findings. Further studies with larger and more balanced cohorts are necessary to validate the diagnostic utility of this method.

**Table 1:** Comparison between the two studied groups according to demographic data

	Cyst (*n* = 8)	Granuloma (*n* = 49)	χ^2^	^FE^ *p*
**Age** **Sex**	34.38 ± 6.301	34.04 ± 8.832	0.919	
Female	4 (50.0%)	26 (53.1%)	0.026	1.000
Male	4 (50.0%)	23 (46.9%)		
**Location**				
Max. ant	3 (37.5%)	22 (44.9%)	0.153	1.000
Max. post	3 (37.5%)	18 (36.7%)	0.002	1.000
Mand. Ant	0 (0.0%)	2 (4.1%)	0.338	1.000
Mand. Post	2 (25.0%)	7 (14.3%)	0.594	0.599

χ^2^: Chi square test FE: Fisher Exact

*p: p value for comparing between the two studied groups*

*: Statistically significant at p ≤ 0.05

**Table 2:** Relation between observer 1 with observer 1 reassessment and observer 2 in total sample (*n* = 57)

	Observer 1		χ^2^	*p*
	Score 1 (*n* = 29)	Score 2 (*n* = 28)		
**Observer 1 reassessment**				
Score 1	29 (100.0%)	1 (5.0%)	53.132^*^	< 0.001^*^
Score 2	0 (0.0%)	27 (96.4%)		
**κ (Level of agreement)**	0.965 (Very good)			
**Observer 2**				
Score 1	28 (96.6%)	1 (3.6%)	49.278^*^	< 0.001^*^
Score 2	1 (3.4%)	27 (96.4%)		
**κ (Level of agreement)**	0.930 (Very good)			

χ^2^: Chi square test κ: kappa test

*p: p value for Relation between Observer 1 with Observer 2*

*: Statistically significant at p ≤ 0.05

**Table 3:** Comparison between the two studied groups according to scoring

Cyst (*n* = 8)	Granuloma (*n* = 49)	χ^2^	^FE^ *p*	
Digital eye				
**Observer 1**				
Score 1	0 (0.0%)	29 (59.2%)	9.638^*^	0.002^*^
Score 2	8 (100.0%)	20 (40.8%)		
**Observer 1 reassessment**				
Score 1	0 (0.0%)	30 (61.2%)	10.340^*^	0.001^*^
Score 2	8 (100.0%)	19 (38.8%)		
**Observer 2**				
Score 1	1 (12.5%)	28 (57.1%)	5.484^*^	0.025^*^
Score 2	7 (87.5%)	21 (42.9%)		

χ^2^: Chi square test FE: Fisher Exact

*p: p value for comparing between the two studied groups*

*: Statistically significant at p ≤ 0.05

## Electronic supplementary material

Below is the link to the electronic supplementary material.

Supplementary Material 1

## Data Availability

Data are available from the corresponding author upon request after approval by all authors.

## References

[1] Juerchott A, Pfefferle T, Flechtenmacher C, Mente J, Bendszus M, Heiland S et al (2018) Differentiation of periapical granulomas and cysts by using dental MRI: a pilot study. Int J Oral Sci 10(2):17 29777107 10.1038/s41368-018-0017-y PMC5966810

[2] Orstavik D (2020) Essential endodontology: prevention and treatment of apical periodontitis. Wiley

[3] Nair PR, Pajarola G, Schroeder HE (1996) Types and incidence of human periapical lesions obtained with extracted teeth. oral surgery, oral medicine, oral pathology, oral radiology, and endodontology. 81(1):93–102 10.1016/s1079-2104(96)80156-9 8850492

[4] Prakash R, Shyamala K, Girish H, Murgod S, Singh S, Rani PV (2016) Comparison of components of odontogenic cyst fluids: A review. J Med Radiol Pathol Surg 2(2):15–17

[5] Etöz M, Amuk M, Avcı F, Yabacı A (2021) Investigation of the effectiveness of CBCT and Gray scale values in the differential diagnosis of apical cysts and granulomas. Oral Radiol 37:109–117 32613300 10.1007/s11282-020-00459-6

[6] Sönmez G, Kamburoğlu K, Yılmaz F, Koç C, Barış E, Tüzüner A (2019) Versatility of high resolution ultrasonography in the assessment of granulomas and radicular cysts: a comparative in vivo study. Dentomaxillofacial Radiol 48(6):20190082 10.1259/dmfr.20190082 PMC6747443 31188679

[7] Aggarwal V, Singla M (2010) Use of computed tomography scans and ultrasound in differential diagnosis and evaluation of nonsurgical management of periapical lesions. oral surgery, oral medicine, oral pathology, oral radiology, and endodontology. 109(6):917–923 10.1016/j.tripleo.2009.12.055 20399691

[8] Fernandes M, de Ataide I (2010) Nonsurgical management of periapical lesions. J Conservative Dentistry Endodontics 13(4):240–245 10.4103/0972-0707.73384 PMC3010029 21217952

[9] Jorge EG, Tanomaru-Filho M, Gonçalves M, Tanomaru JM (2008) Detection of periapical lesion development by conventional radiography or computed tomography. oral surgery, oral medicine, oral pathology. Oral Radiol Endodontology 106(1):e56–e61 10.1016/j.tripleo.2008.03.020 18585613

[10] Guo J, Simon JH, Sedghizadeh P, Soliman ON, Chapman T, Enciso R (2013) Evaluation of the reliability and accuracy of using cone-beam computed tomography for diagnosing periapical cysts from granulomas. J Endod 39(12):1485–1490 24238434 10.1016/j.joen.2013.08.019

[11] Okada K, Rysavy S, Flores A, Linguraru MG (2015) Noninvasive differential diagnosis of dental periapical lesions in cone-beam CT scans. Med Phys 42(4):1653–1665 25832055 10.1118/1.4914418

[12] Chanani A, Adhikari HD (2017) Reliability of cone beam computed tomography as a biopsy-independent tool in differential diagnosis of periapical cysts and granulomas: an: in vivo: study. J Conservative Dentistry 20(5):326–331 10.4103/JCD.JCD_124_17 PMC5767827 29386780

[13] Künzel A, Lehmann T, Benz C, Schmitt W, Kaser A (1995) Colouring digital dental radiographs. Dentomaxillofacial Radiol 2(24):100–101

[14] Shi XQ, Li G, Yoshiura K, Welander U (2004) Perceptibility curve test for conventional and colour-coded radiographs. Dentomaxillofacial Radiol 33(5):318–322 10.1259/dmfr/27372105 15585809

[15] Booshehry MZ, Davari A, Ardakani FE, Nejad MRR (2010) Efficacy of application of pseudocolor filters in the detection of interproximal caries. J Dent Res Dent Clin Dent Prospects 4(3):79 22991603 10.5681/joddd.2010.020 PMC3429980

[16] Davari A, Booshehri M, Ezoddini F, Danesh Kazemi A, Mousavinasab M, Rashidinejad M (2010) The efficacy of application of pseudocolor software in digital radiography for detection of interproximal caries. J Dent 11(3):215–221

[17] Mehralizadeh S, Mohammadi S, Talaeepour A, Mehrvarzfar P, Mirzaee M (2014) Diagnostic accuracy of digital phosphor plates using Pseudo-Color enhancement for detection of horizontal root fractures in Single-Rooted teeth. J Iran Dent Association 26(3):177–183

[18] Bräger U, Bürgin W, Marconi M, Häsler R, Lang N (1994) Influence of contrast enhancement and pseudocolor transformation on the diagnosis with digital Subtraction images (DSI). J Periodontal Res 29(2):95–102 8158504 10.1111/j.1600-0765.1994.tb01096.x

[19] ŞATIR S, Cimen T, GÜLŞEN İT (2023) SAKA YY. Diagnosing pathological changes in the Non-thickening sinus mucosa: A retrospective CBCT study with Psudocolor imaging. Bezmialem Sci.;11(3)

[20] Uraba S, Ebihara A, Komatsu K, Ohbayashi N, Okiji T (2016) Ability of cone-beam computed tomography to detect periapical lesions that were not detected by periapical radiography: a retrospective assessment according to tooth group. J Endod 42(8):1186–1190 27372162 10.1016/j.joen.2016.04.026

[21] Moyano M (2012) The pseudocolor in digital radiographic image. Diagnosis of demineralization in dental calcified tissues

[22] Nikneshan S, Boroumand R, Esmaeeli N, Azadikhah A, Paknahad M (2023) The effect of different image processing techniques on the measurement accuracy of endodontics file length. J Dent 24(3):335 10.30476/dentjods.2022.95708.1885 PMC10506146 37727355

