# Supplementary figures and images for: Pseudo-color enhancement of CBCT images as a diagnostic tool for radicular cysts and periapical granulomas: A clinical feasibility study
Source: Clin Oral Investig. 2025 Jul 3;29(7):371. doi: 10.1007/s00784-025-06425-y (PMC12226692; doi:10.1007/s00784-025-06425-y)

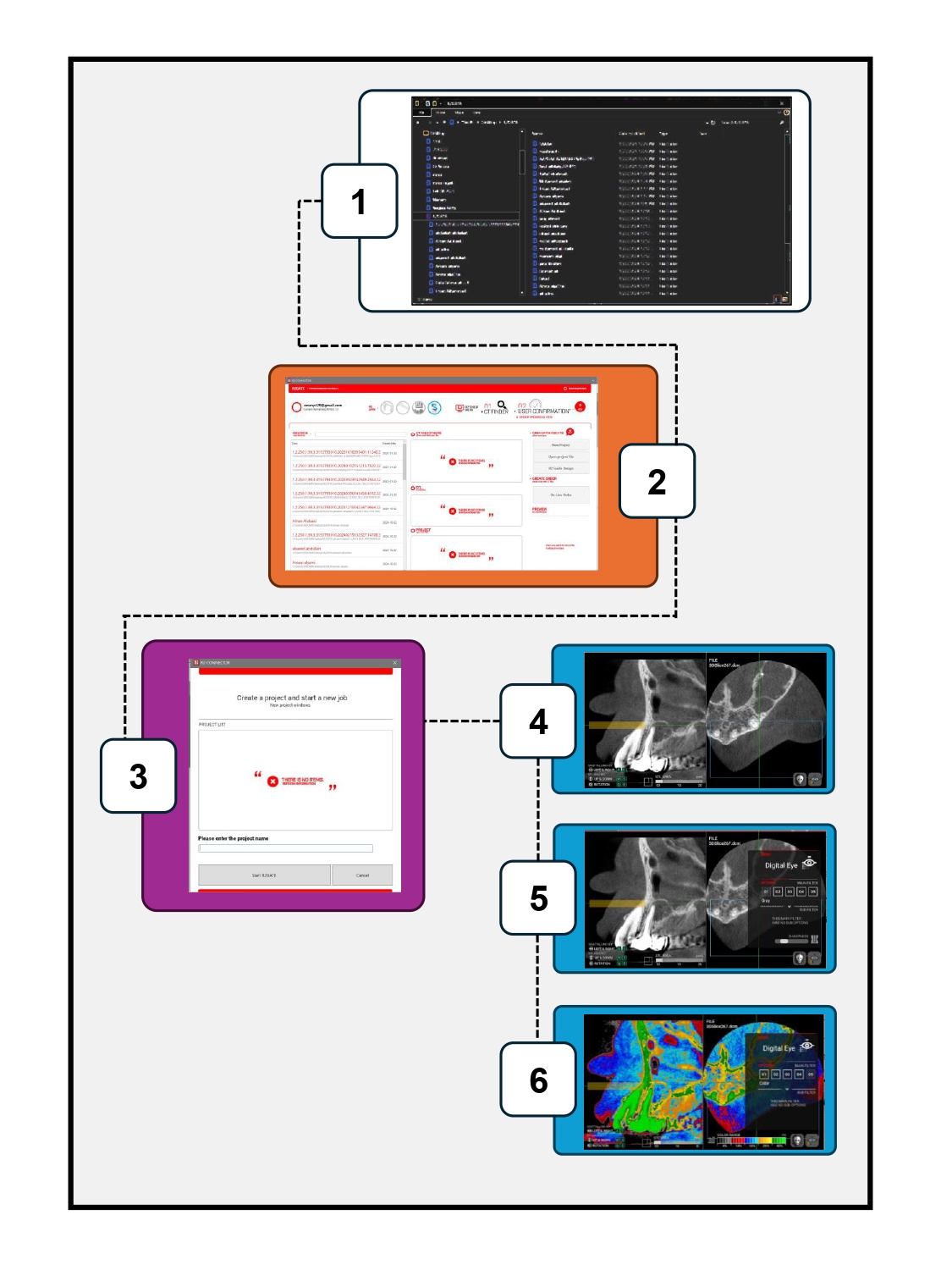

Supplement: Supplementary file 1 — Supplementary Material 1 [file 784_2025_6425_MOESM1_ESM.jpg]
